# Involvement of sulfated biopolymers in adhesive secretions produced by marine invertebrates

**DOI:** 10.1242/bio.037358

**Published:** 2018-09-20

**Authors:** Elise Hennebert, Edwicka Gregorowicz, Patrick Flammang

**Affiliations:** 1 Cell Biology Unit, Research Institute for Biosciences, University of Mons, 23 Place du Parc, 7000 Mons, Belgium; 2 Biology of Marine Organisms and Biomimetics Unit, Research Institute for Biosciences, University of Mons, 23 Place du Parc, 7000 Mons, Belgium

**Keywords:** Sulfate, Marine adhesion, Mussel, Tubeworm, Sea star, Limpet, Sea cucumber

## Abstract

Many marine invertebrates use adhesive secretions to attach to underwater surfaces and functional groups borne by their adhesive proteins and carbohydrates, such as catechols and phosphates, play a key role in adhesion. The occurrence of sulfates as recurrent moieties in marine bioadhesives suggests that they could also be involved. However, in most cases, their presence in the adhesive material remains speculative. We investigated the presence of sulfated biopolymers in five marine invertebrates representative of the four types of adhesion encountered in the sea: mussels and tubeworms for permanent adhesion, limpets for transitory adhesion, sea stars for temporary adhesion and sea cucumbers for instantaneous adhesion. The dry adhesive material of mussels, sea stars and sea cucumbers contained about 1% of sulfate. Using anti-sulfotyrosine antibodies and Alcian Blue staining, sulfated proteins and sulfated proteoglycans and/or polysaccharides were identified in the secretory cells and adhesive secretions of all species except the tubeworm. Sulfated proteoglycans appear to play a role only in the non-permanent adhesion of sea stars and limpets in which they could mediate cohesion within the adhesive material. In mussels and sea cucumbers, sulfated biopolymers would rather have an anti-adhesive function, precluding self-adhesion.

## Abstract (precis)

**Summary:** Sulfate groups borne by adhesive macromolecules play a role in attachment to underwater surfaces in marine invertebrates using non-permanent adhesion, but not in those using permanent or instantaneous adhesion.

## INTRODUCTION

Many marine organisms, ranging from microscopic bacteria and algae to macroscopic seaweeds and invertebrates, use adhesive secretions to attach to underwater surfaces (Walker, 1987; von Byern and Grunwald, 2010; Smith, 2016a). Adhesive systems are particularly developed and diversified in marine invertebrates and they may differ considerably in their mode of operation, their structure and the composition of their adhesive secretions (Smith, 2016a). Different types of adhesion can therefore be distinguished (Tyler, 1988; Whittington and Cribb, 2001; Flammang et al., 2005). Permanent adhesion involves the secretion of an adhesive that hardens with time and forms a durable cement. Non-permanent adhesion allows simultaneous adhesion and locomotion. Some organisms creep on a viscous film they produce and leave behind them as they move (transitory adhesion). Others attach firmly but only temporarily to the substratum, being able to attach and detach repetitively (temporary adhesion). Finally, instantaneous adhesion relies on single-use organs or cells, and is used in functions other than attachment to the substratum requiring a very fast formation of adhesive bonds. Different types of biopolymers are usually observed in both temporary and permanent bioadhesives. These include proteins, glycoproteins and polysaccharides, as well as sulfated and phosphorylated versions of these polymers (Smith, 2016a). To function effectively as a holdfast, marine adhesives must possess several characteristics such as the ability to displace water and hydration layers from the substratum, spread and rapidly form strong adhesive bonds with the surface and, in sessile organisms, the ability to cure and resist microbial degradation (Waite, 1987; Kamino, 2010). These characteristics derive from the physicochemical properties of the adhesive proteins and carbohydrates and, in particular, from the functional groups they bear (Sagert et al., 2006; Flammang et al., 2009; Petrone, 2013). In marine adhesives, these groups are of three main types: catechol, basic (amines, guanidinium or imidazole) and acidic (carboxylates, phosphates or sulfates) (Stewart et al., 2011; Petrone, 2013). Among these functional groups, catechols, amines and phosphates have been the most investigated and appear to play a key role in the adhesion of marine invertebrates.

Catechols are present in the adhesive proteins of mussels and tubeworms in the form of DOPA, a residue formed by the post-translational hydroxylation of tyrosine residues. This modified amino acid can bind to mineral surfaces either through hydrogen bonds or by forming coordination complexes with metal ions and metal oxides (Lee et al., 2006, 2011; Sagert et al., 2006; Waite, 2017). It is also involved in the formation of cross-links between proteins, thereby contributing to the cohesive strength of the adhesive material. Mussel and tubeworm adhesive proteins are also particularly rich in amine-bearing lysine residues (Stewart et al., 2011, 2017). The adsorption of amines onto mineral oxide surfaces and biofilms, which are both negatively charged at neutral pH values, appears to take place primarily via electrostatic interactions (Stewart et al., 2011). Moreover, it was demonstrated recently that lysine and DOPA residues act synergistically to provide surface adhesion in seawater: the amine groups displace hydrated cations from the mineral surface, allowing the catechol groups to bind to underlying oxides (Maier et al., 2015). Phosphate is found in the adhesive proteins of brown algal spores, mussels, tubeworms and sea cucumbers as phosphoserine, a monoester-phosphate resulting from the post-translational modification of serine residues (Zhao et al., 2005; Silverman and Roberto, 2007; Flammang et al., 2009). Phosphoserine residues are thought to contribute both cohesive (by Ca^2+^ bringing) and adhesive roles to these glues (Zhao and Waite, 2006; Sun et al., 2007). Strong adsorption of phosphate moieties to metal oxide surfaces occurs through complexation or electrostatic interaction (Stewart et al., 2011; Petrone, 2013).

The occurrence of sulfates as recurrent moieties in marine bioadhesives suggests that they could also play a role in the adhesion of the organisms producing them (Petrone, 2013). Indeed, such functional groups have been described in the adhesive secretions produced by macrophytic algae (Tarakhovskaya, 2014), planarians (Hayes, 2017), gastropod molluscs (Grenon and Walker, 1978, 1980; Bravo-Portela et al., 2012; Petraccioli et al., 2013), tubeworms (Wang and Stewart, 2013) and sea stars (Engster and Brown, 1972; Perpeet and Jangoux, 1973; Flammang et al., 1998). However, in most cases, they have only been detected histochemically in gland cells and their presence in the adhesive material remains speculative. Moreover, the nature of the molecules bearing the sulfate groups is not known with certainty. Sulfation involves the transfer of a sulfate moiety from a donor co-substrate to a hydroxyl or amino group of a substrate molecule. Substrates can be proteins, glycoproteins, proteoglycans, polysaccharides or glycolipids (Hemmerich, 2007; Pomin, 2009). Protein sulfation is a post-translational modification generally occurring on tyrosine residues, although some cases of serine and threonine sulfation have been reported (Medzihradszky et al., 2004). Sulfated proteins are generally secreted or incorporated into the plasma membrane (Monigatti et al., 2006). They include adhesion molecules, coagulation factors, G-protein-coupled receptors, hormone receptors, proteins of the extracellular matrix (ECM) and immune components (Woods et al., 2007; Kanan and Al-Ubaidi, 2013). In many of these proteins, the sulfate group improves their ability to interact with other proteins (Kehoe and Bertozzi, 2000; Monigatti et al., 2006; Kanan and Al-Ubaidi, 2013). Sulfated carbohydrates can be found in polysaccharides (e.g. sulfated fucans and galactans secreted by macroalgae and invertebrates) or within glycoconjugates such as proteoglycans (e.g. heparan sulfate proteoglycans of the ECM) (Hemmerich, 2007; Pomin, 2009). In the latter, sulfates are binding sites for growth and differentiation factors, adhesion molecules and chemoattractants (Hemmerich, 2007). In the defensive secretion of the terrestrial slug *Arion subfuscus*, heparan-sulfate-like proteoglycans form a large, tangled network that gives toughness to the glue (Wilks et al., 2015).

**Fig. 1.**
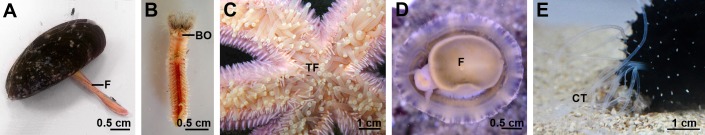
**Model organisms used in this study and their adhesive organs.** (A) The mussel *M. edulis*. (B) The tubeworm *S. alveolata*. (C) The sea star *A. rubens* (oral view). (D) The limpet *P. vulgata* (ventral view). (E) The sea cucumber *H. forskali* (posterior part). BO, building organ; CT, Cuvierian tubules; F, foot; TF, tube feet.

As demonstrated by the above examples, sulfates appear as key functionalities to provide or improve interactions between different molecules and thus could be involved in adhesive mechanisms. In this study, we investigated the presence of sulfated biopolymers in five marine invertebrates representative of the four types of adhesion encountered in the sea (Flammang et al., 2005, 2016): the mussel *Mytilus edulis* and the tubeworm *Sabellaria alveolata* for permanent adhesion, the limpet *Patella vulgata* for transitory adhesion, the sea star *Asterias rubens* for temporary adhesion and the sea cucumber *Holothuria forskali* for instantaneous adhesion (Fig. 1). Total sulfate content was assayed in the adhesive material of three of these species. We used anti-sulfotyrosine antibodies to investigate the presence of sulfated proteins in the adhesive secretions produced by the different organisms as well as to localize the cells producing them in the adhesive organs. Alcian Blue staining was also performed on histological sections and adhesive prints to highlight the presence of sulfated polysaccharides or glycoconjugates.

## RESULTS

### Sulfate content of the adhesive secretions

The benzidine method was used to estimate the sulfate content of the adhesive plaques of *M. edulis*, the footprints of *A. rubens*, and the Cuvierian tubule prints of *H. forskali*. Results are presented in Table 1 together with the results found in the literature for *P. vulgata* and *A. rubens* (Grenon and Walker, 1980; Flammang et al., 1998; respectively). In the mussel, the sulfate content amounted to 1.4% of the adhesive plaque dry mass. For the sea star, the content measured in the present study, 1.15%, was half the quantity measured by Flammang et al. (1998), a difference which is presumably linked to the difficulty to weigh accurately a tiny mass of dry footprint material. In the sea cucumber, we found three times more sulfate in whole print material than in glue-enriched print material (1.55% and 0.53%, respectively). The sulfate content of the cement of *S. alveolata* was not investigated because it was not possible to estimate the starting mass of cement material.

**Table 1.**
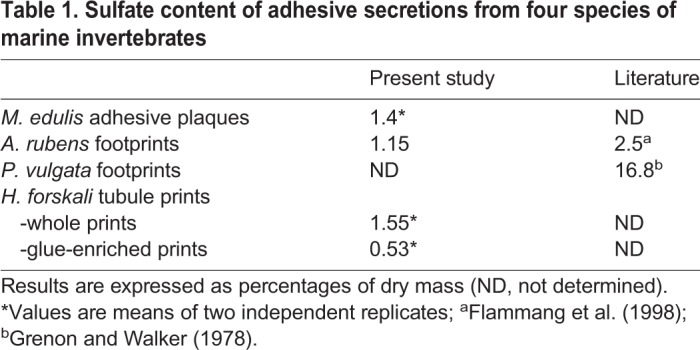
**Sulfate content of adhesive secretions from four species of marine invertebrates**

### Detection of sulfated biopolymers in adhesive organs and secretions

The presence of sulfated macromolecules was investigated in the adhesive organs and adhesive secretions of *M. edulis*, *S. alveolata*, *A. rubens*, *P. vulgata* and *H. forskali*. Sulfated biopolymers were investigated using Alcian Blue staining. Alcian Blue is a cationic dye commonly used in histochemistry to stain carbohydrate-containing molecules (Bancroft and Gamble, 2001). Its affinity for functional groups is pH-dependent: at low pH (<1), it is specific for sulfated molecules, sulfates being the only ionized groups; while, at higher pH (>2.5), carboxylates are also ionized and both types of groups carry a negative charge (Bancroft and Gamble, 2001). Methylation and saponification reactions were also performed before treatments with Alcian Blue at pH 2.5. Methylation converts carboxylates to methyl esters and hydrolyses N- and O-sulfates, leading to a complete loss of Alcian Blue reactivity. Saponification, by cleaving the bonds of methyl esters formed during methylation, allows the restoration of the carboxylates only (Bancroft and Gamble, 2001). Regarding sulfated proteins, they were investigated using anti-sulfotyrosine antibodies, tyrosine being the main amino acid subjected to sulfation (Medzihradszky et al., 2004).

#### Mussels

To secure themselves to the substratum, mussels produce an extra-organismic holdfast, the so-called byssus (Waite, 1983, 2017). The byssus consists of a bunch of proteinaceous filaments connecting the animal to the substratum. Each filament is made up of a proximal thread, functioning as a mooring line, and a distal attachment plaque, securing the filament on the substratum.

**Fig. 2.**
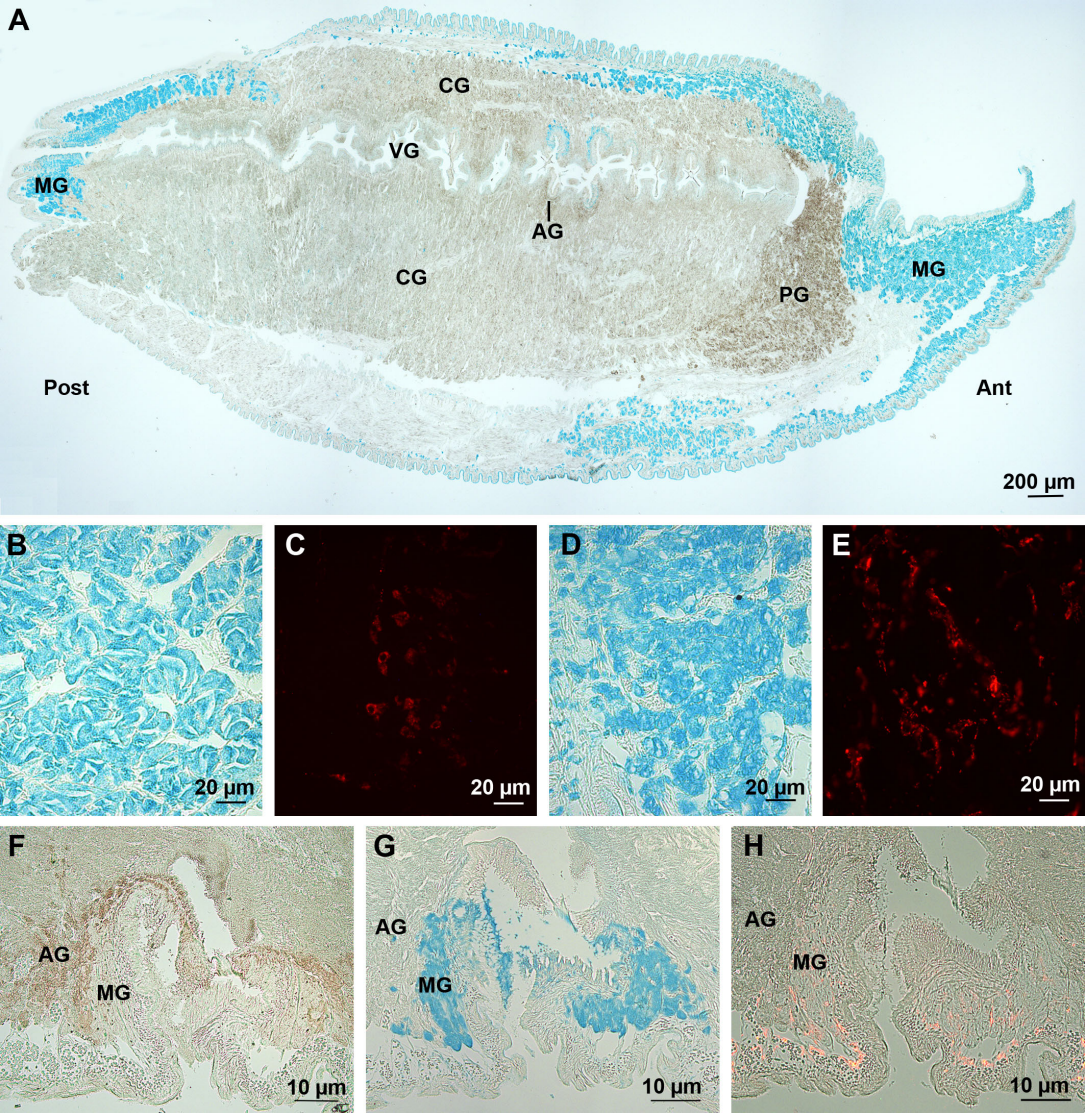
**Sulfated biopolymers in the foot of *M. edulis*.** (A) Sagittal section stained with Alcian Blue at pH 1. (B,C) Anterior mucous cells stained with Alcian Blue at pH 1 and anti-sulfotyrosine antibodies, respectively. (D,E) Posterior mucous cells stained with Alcian Blue at pH 1 and anti-sulfotyrosine antibodies, respectively. (F-H) Transverse section through the middle part of the foot showing the accessory gland stained using Arnow stain (F) and the mucous cells stained with Alcian Blue at pH 1 (G) and anti-sulfotyrosine antibodies (H). AG, accessory gland; Ant, anterior; CG, collagen gland; MG, mucous gland; PG, phenol gland; Post, posterior; VG, ventral groove.

Mussel byssal filaments are produced by the foot of the animal (Fig. 1A), their different constituting proteins being secreted and assembled into a groove running along its ventral side. Three glands, distributed along the foot, contribute to the formation of the filaments: the phenol gland, the accessory gland, and the collagen gland (Priemel et al., 2017; Waite, 2017; Fig. 2A). In addition, a fourth gland, the mucous gland, has been described but whether or not it participates to byssus formation remains unknown (Pujol, 1967; Waite, 1983). Using Alcian Blue at pH 1, an intense blue staining was observed for the mucous glands present in the foot anterior and posterior parts as well as along the groove (Fig. 2A,B,D,G), highlighting the presence of highly sulfated molecules in these glands. The same glands were labelled in immunohistochemistry using anti-sulfotyrosine antibodies, although the labelling appeared weaker (Fig. 2C,E,H). Regarding the labelled cells present around the groove, they are clearly different from those of the accessory gland, which enclose DOPA-containing proteins and are therefore reactive with Arnow stain (Fig. 2F-H). Using Alcian Blue at pH 2.5, an additional faint staining of the collagen gland was observed (Table 2). This reactivity is lost after methylation, but almost completely restored after saponification (Table 2), indicating the presence of carboxylated molecules in mucous and collagen glands.

**Table 2.**
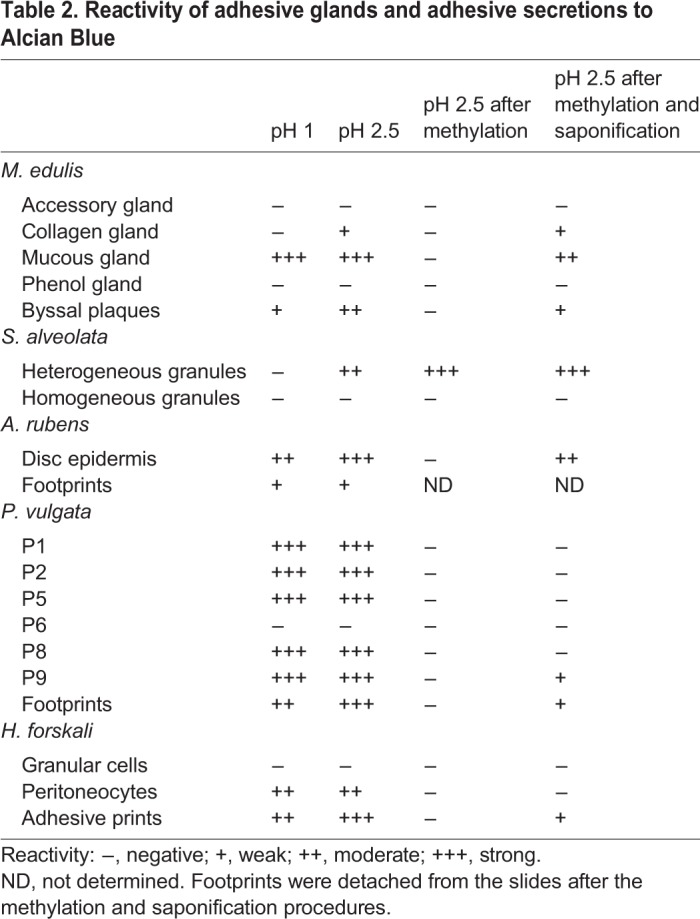
**Reactivity of adhesive glands and adhesive secretions to Alcian Blue**

**Fig. 3.**
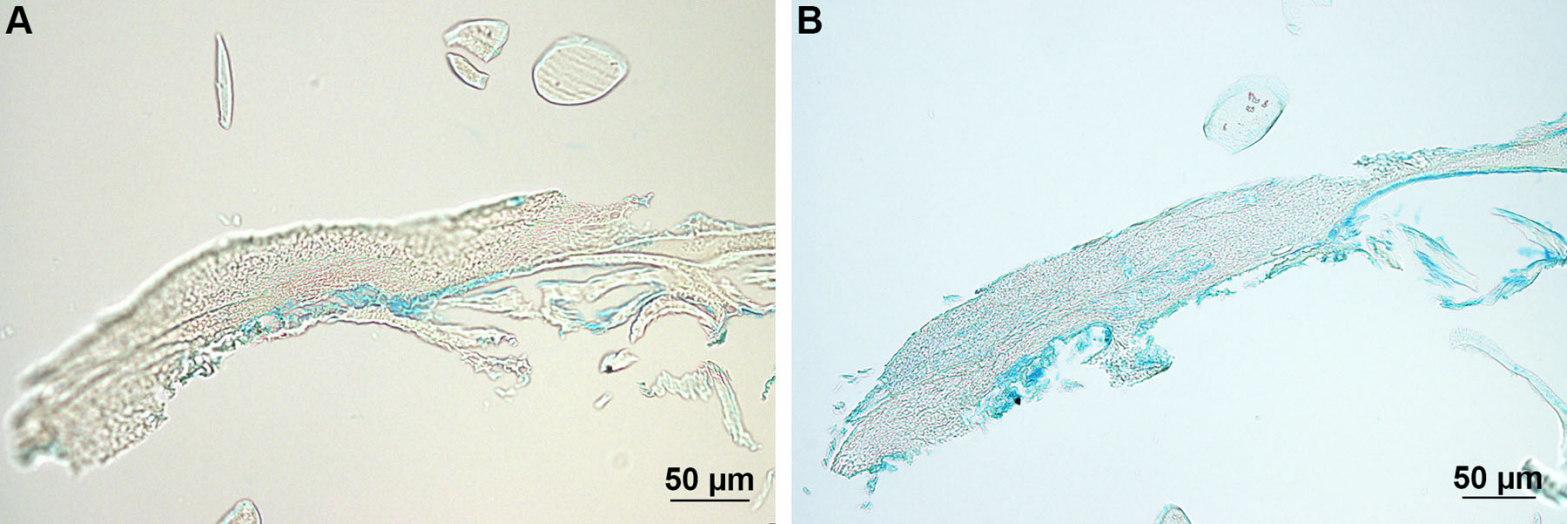
**Byssal adhesive plaque of *M. edulis* stained with Alcian Blue.** (A) Staining at pH 1. (B) Staining at pH 2.5.

Byssal plaques were stained with Alcian Blue at both pH 1 and 2.5 (Fig. 3), while no specific immunolabelling was observed using anti-sulfotyrosine antibodies. The Alcian Blue staining, however, was restricted to the edge (i.e. cuticle) of the plaque and thread (Fig. 3).

#### Tubeworms

**Fig. 4.**
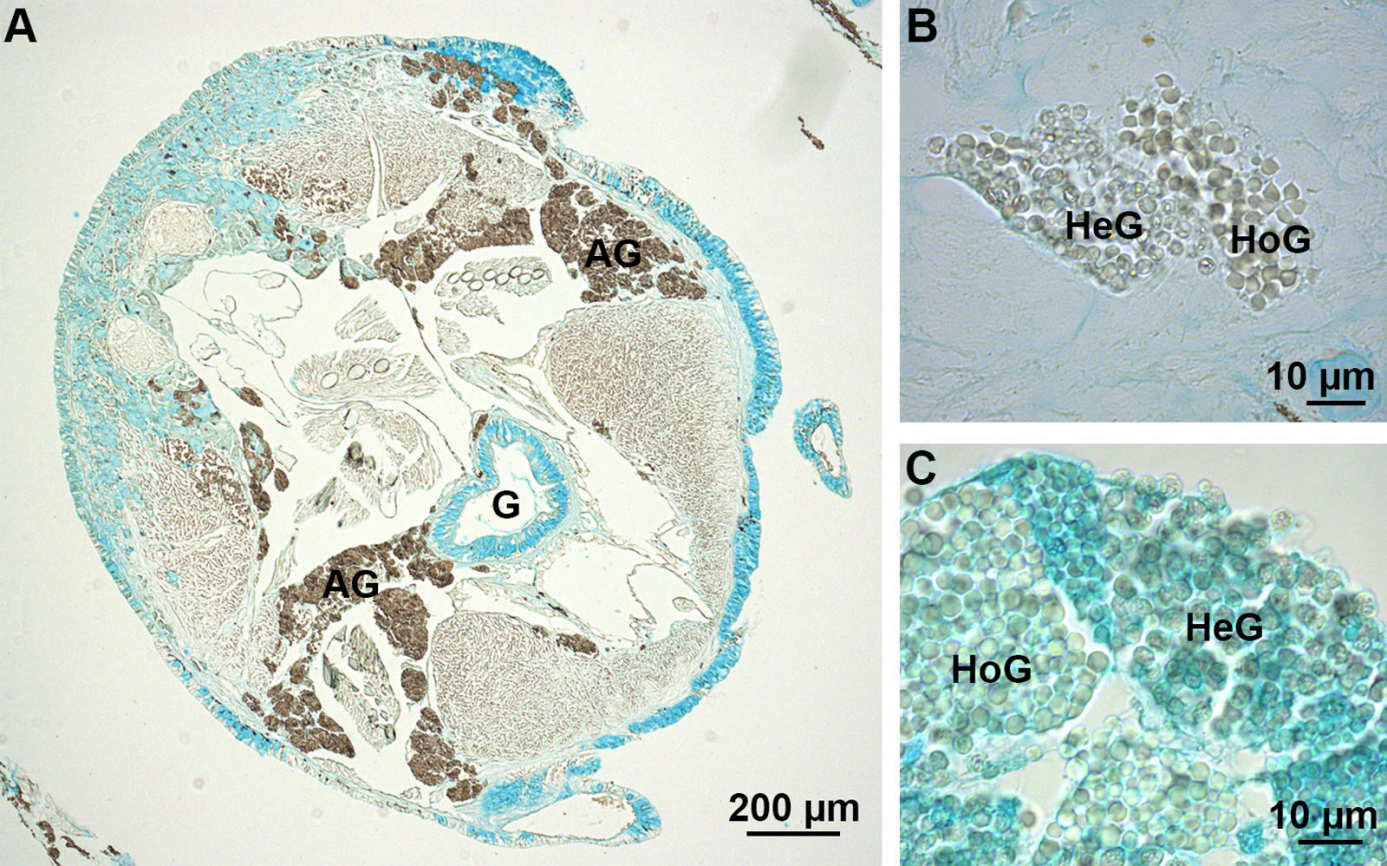
**Absence of sulfated biopolymers in the adhesive glands of *S. alveolata*.** (A) Transverse section in the thorax stained with Alcian Blue at pH 1. (B,C) Cement glands stained with Alcian Blue at pH 1 and 2.5, respectively. AG, adhesive glands; G, gut; HeG, heterogeneous granules; HoG, homogeneous granules.

Sabellariids are tube-dwelling marine polychaetes that live in the intertidal zone. To build their tube, they collect sand grains or mollusc shell fragments in their surroundings, dab them with spots of cement, and assemble them into a rigid composite tube (Hennebert et al., 2015; Stewart et al., 2017). This cement is secreted by the so-called building organ, a complex secretory organ made up of bouquets of cement cells located deep within the thorax of the worms (Fig. 1B). Two types of cement cells have been described in the literature, which contain respectively homogeneous and heterogeneous secretory granules (Becker et al., 2012; Stewart et al., 2017). In the species *Phragmatopoma californica*, Wang and Stewart (2013) described the presence of sulfated polysaccharides in the homogeneous secretory granules using Alcian Blue staining and elemental analysis. In *S. alveolata*, however, no staining was observed in any of the two types of cells using Alcian Blue at pH 1 (Fig. 4A,B). At pH 2.5, the heterogeneous granules were stained while no staining was observed for the homogeneous granules (Fig. 4C). Interestingly, the reactivity of heterogeneous granules with Alcian Blue at pH 2.5 was not lost when the sections were submitted to methylation (Table 2), indicating that groups other than carboxylates are responsible for the reactivity. Heterogeneous granules are known to contain polyphosphoproteins (Becker et al., 2012; Wang and Stewart, 2012) and their staining with Alcian Blue at pH 2.5 could therefore highlight the presence of the phosphate groups. No specific labelling of the cement cells was observed using the anti-sulfotyrosine antibody (results not illustrated).

#### Sea stars

Asteroids adhere firmly to various substrata thanks to adhesive secretions released by their tube feet (Fig. 1C). Adhesion is temporary, however, and after the tube foot has become voluntarily detached, the adhesive material remains firmly bound to the substratum as a footprint (Flammang et al., 2016).

**Fig. 5.**
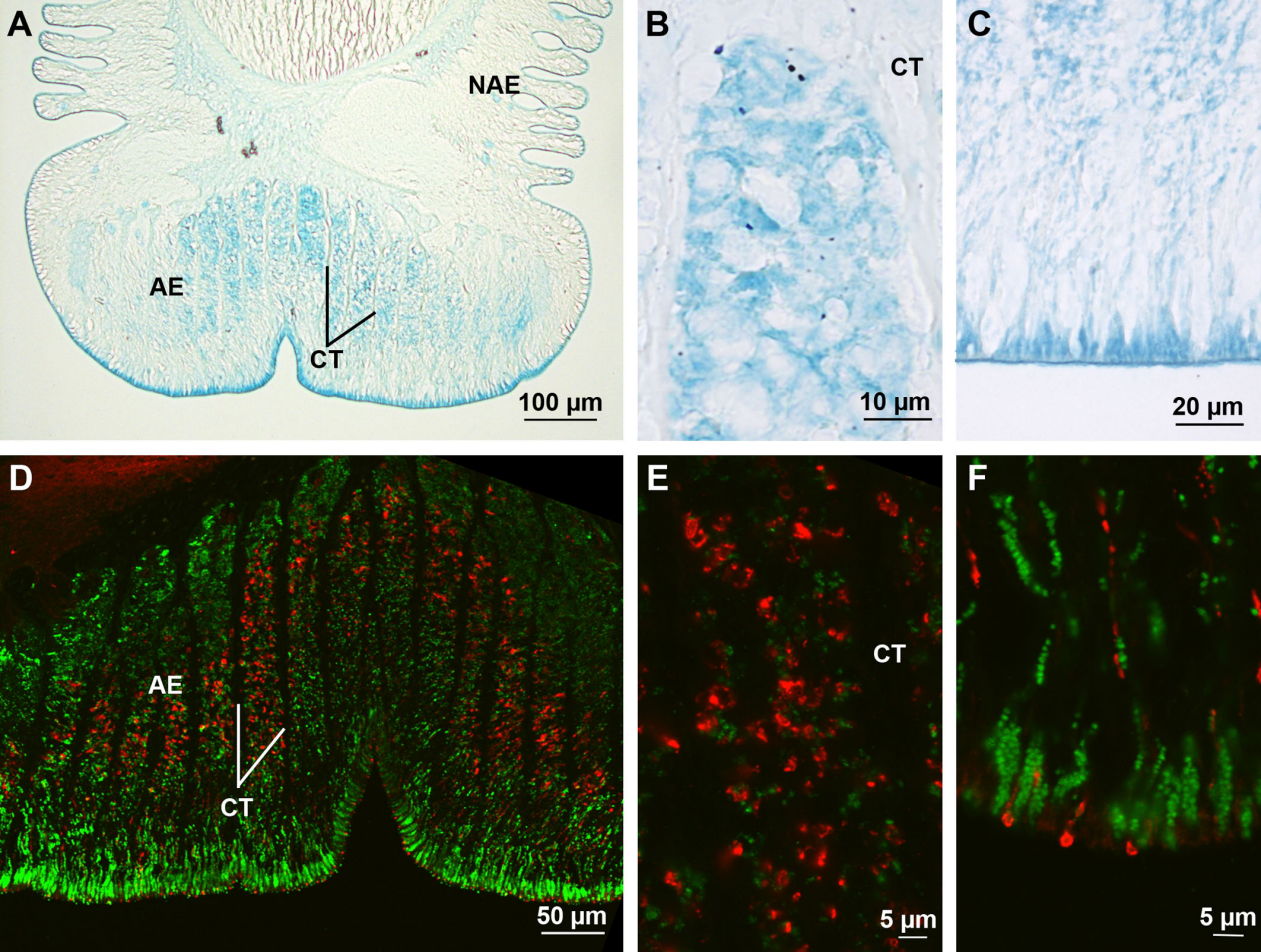
**Sulfated biopolymers in the tube foot disc of *A. rubens*.** (A) Longitudinal section through a tube foot stained with Alcian Blue at pH 1. (B,C) Details of (A) in the basal and apical parts of the adhesive epidermis, respectively. (D) Longitudinal section through a disc immunolabelled with anti-sulfotyrosine (red) and anti-Sfp1 (green) antibodies. (E,F) Details of (D) in the middle part of adhesive epidermis and at the level of the disc surface, respectively. AE, adhesive epidermis; CT, connective tissue; NAE, non-adhesive epidermis.

In *A. rubens*, the distal part of tube feet, the disc, is made up of a thick adhesive epidermis reinforced by connective tissue septa (Fig. 5A,C). This epidermis encloses a duo-gland adhesive system comprising both adhesive and de-adhesive cells as well as sensory cells and support cells (Flammang et al., 1994, 1998). A specific staining was observed in the adhesive epidermis with Alcian Blue at pH 1, in cells corresponding in size, shape and disposition to the adhesive cells (Fig. 5A-C). At pH 2.5, most of the disc tissue layers were stained. This reactivity was completely lost after methylation, and restored when methylation was followed by saponification, demonstrating the presence of carboxylated molecules in the disc tissues (Table 2). Alcian Blue staining confirms earlier studies reporting that adhesive cells in sea stars contain both sulfated and carboxylated mucopolysaccharides (Engster and Brown, 1972; Perpeet and Jangoux, 1973). Using the anti-sulfotyrosine antibodies, a specific labelling was observed at the level of the adhesive epidermis (Fig. 5D-F), but its distribution appeared different from that of the staining with Alcian Blue at pH 1. Co-labelling was therefore performed with antibodies directed against Sfp1, the first adhesive protein characterized in sea stars (Hennebert et al., 2014). This method shows that the sulfated proteins are not localized in the adhesive cells (Fig. 5D,F). The narrow shape of anti-sulfotyrosine positive cells suggests they could correspond to de-adhesive or sensory cells (Flammang et al., 1994).

**Fig. 6.**
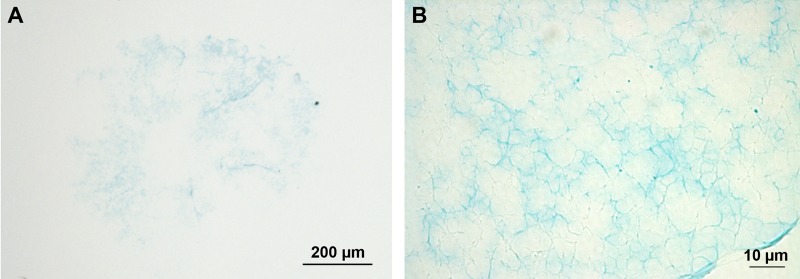
**Adhesive footprint of *A. rubens* stained with Alcian Blue at pH 1.** (A) General view of a footprint. (B) Detail of the footprint structural meshwork.

The adhesive secretion left on the substratum as a footprint after tube foot detachment is composed of a fibrillar meshwork deposited on a homogeneous layer (Hennebert et al., 2008). The meshwork was weakly stained with Alcian Blue at both pH 1 (Fig. 6) and 2.5. No footprints were observed after methylation, suggesting that this treatment would be responsible of their detachment from the glass slides (Table 2). No immunolabelling was observed with the anti-sulfotyrosine antibodies (results not illustrated).

#### Limpets

**Fig. 7.**
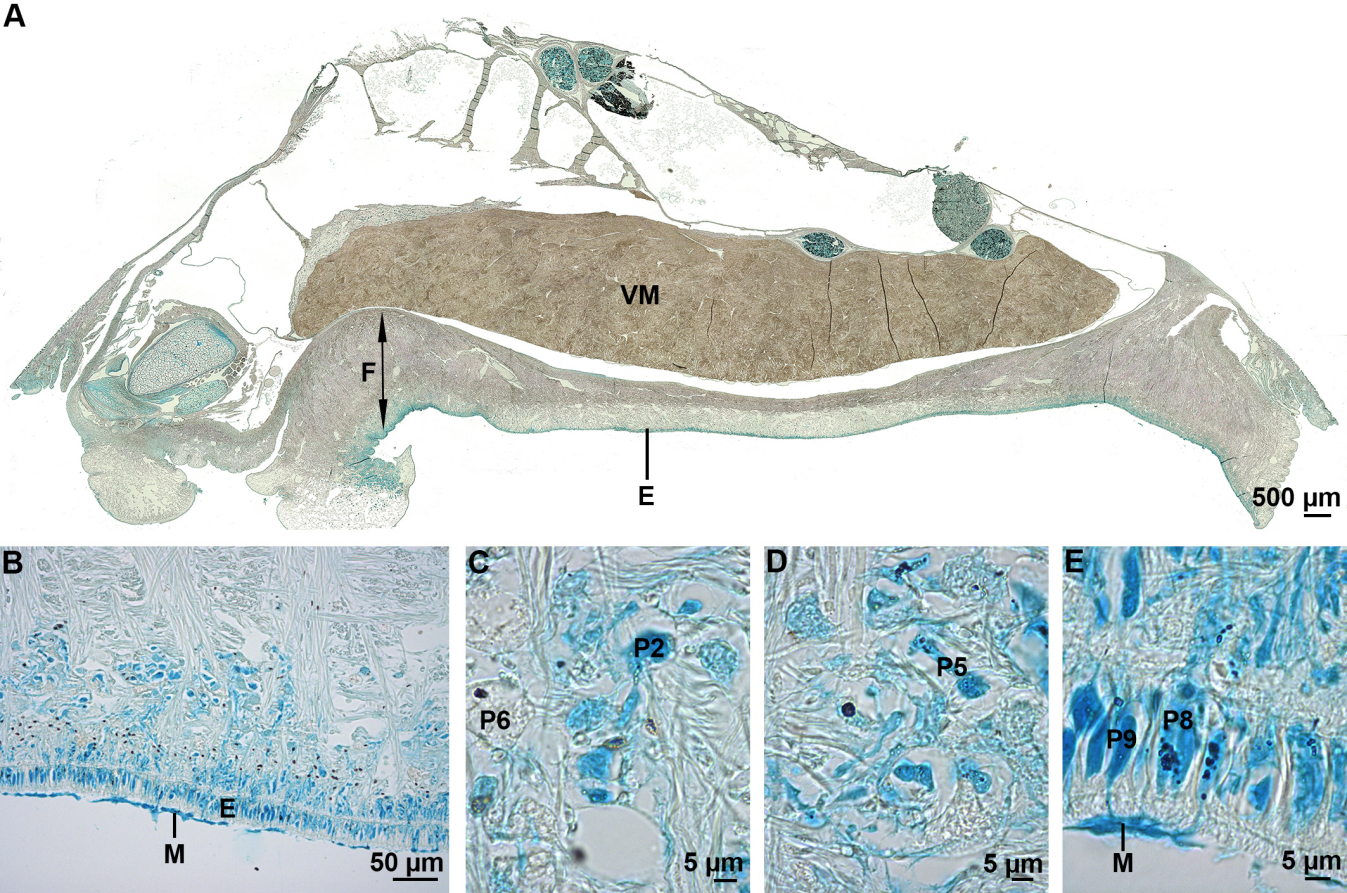
**Sulfated biopolymers in the foot of the limpet *P. vulgata*.** (A) Longitudinal section through the foot and visceral mass of an individual stained with Alcian Blue at pH 1. (B) Higher magnification view showing the sole epithelium and the subepithelial region. (C-E) Detailed views showing the glands P2, P5 and P6 in the subepithelial region, and the glands P8 and P9 in the epithelium. E, epidermis; F, foot; M, mucus; VM, visceral mass.

These gastropod molluscs are well known for their ability to attach tenaciously to rocks in the wave-swept intertidal zone (Grenon and Walker, 1978, 1981; Smith et al., 1999). The limpet foot (Fig. 1D) comprises a complex pedal glandular system involved in the production of mucous secretions with different functions (Grenon and Walker, 1978). In *P. vulgata*, six glands (P1, P2, P5, P6, P8 and P9) would be involved in the secretion of the mucus used for locomotion and adhesion (Grenon and Walker, 1978). The gland P1 is located in the anterior part of the foot, next to the marginal groove. Glands P2, P5, P6, P8 and P9 are scattered all over the foot sole. All possess sub-epithelial cell bodies sending long necks that open between the epidermal cells of the sole, except glands P9 which are intra-epithelial (Fig. 7) (Grenon and Walker, 1978). An intense blue stain was observed at the level of the sole epithelium and of the subepithelial region with Alcian Blue at both pH 1 and 2.5 (Fig. 7B, Table 2). High magnification images show that the glands P1, P2, P5, P8 and P9 were stained, while the glands P6 were not (Fig. 7C-E). The mucus covering the epithelium was also stained with Alcian Blue (Fig. 7B,E). The staining with Alcian Blue was completely lost after methylation and restored partially only in glands P9 when methylation was followed by saponification, indicating that all the glands enclose sulfated macromolecules and that glands P9 also enclose carboxylated molecules (Table 2). No immunolabelling of the sole epithelium and of the subepithelial region was observed using the anti-sulfotyrosine antibodies (results not illustrated).

**Fig. 8.**
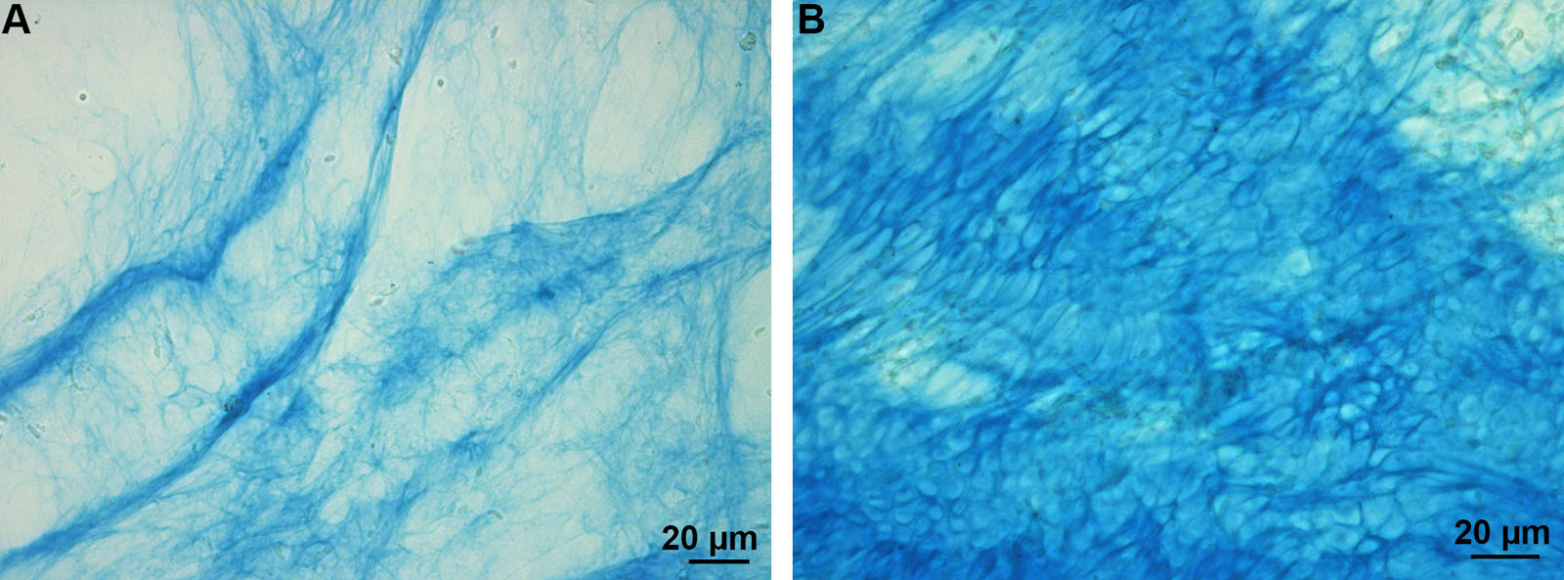
**Adhesive footprints of *P. vulgata* stained with Alcian Blue.** (A) Staining at pH 1. (B) Staining at pH 2.5.

The adhesive footprints left by limpets after they were removed from the glass slides were moderately stained with Alcian Blue at pH 1 and intensively at pH 2.5 (Fig. 8, Table 2). No staining was observed after methylation while a weak staining was restored after saponification (Table 2).

#### Sea cucumbers

**Fig. 9.**
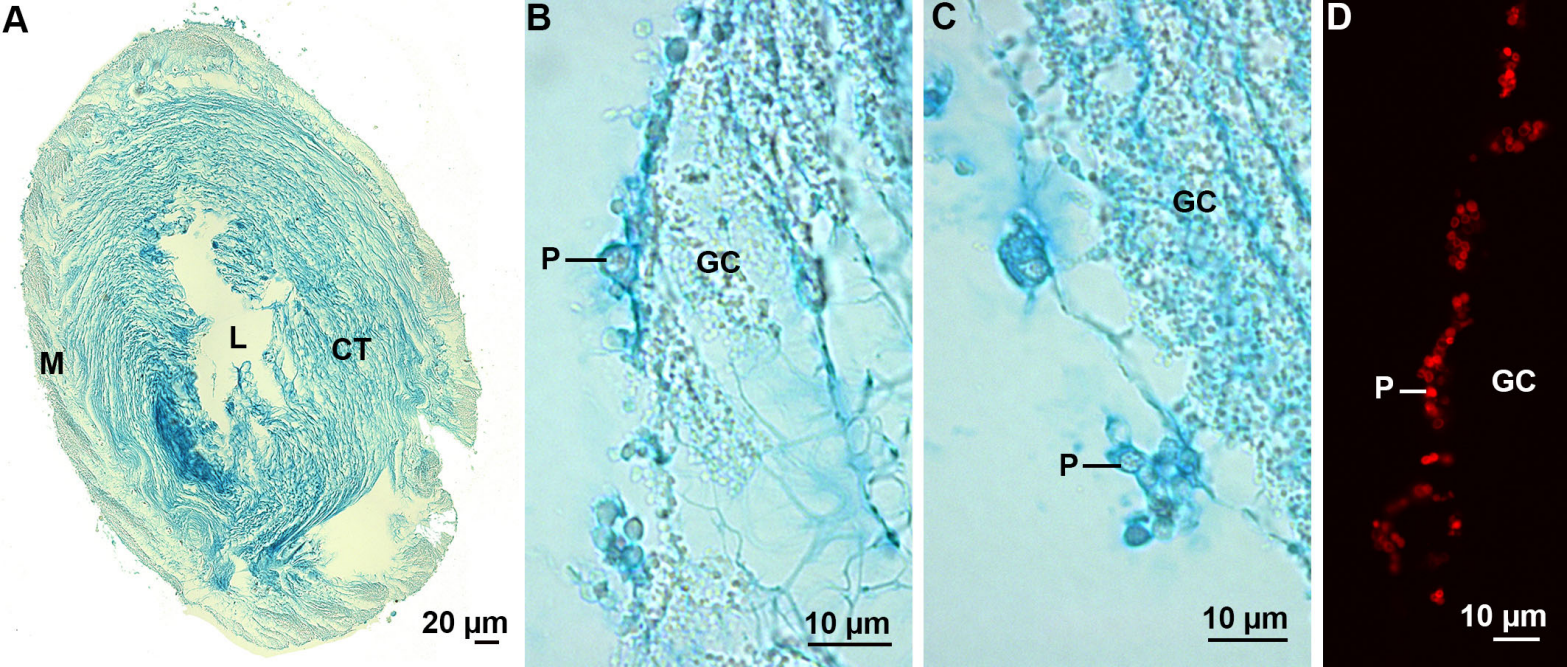
**Sulfated biopolymers in the Cuvierian tubules of the sea cucumber *H. forskali*.** (A) Transverse section through a Cuvierian tubule stained with Alcian Blue at pH 1. (B-D) High magnification images of the mesothelium stained with Alcian Blue at pH 1 (B) and 2.5 (C), and immunolabelled with anti-sulfotyrosine antibodies (D). CT, connective tissue; GC, granular cells; L, lumen; M, mesothelium; P, peritoneocyte.

Cuvierian tubules are present in several species of sea cucumbers in which they occur in great numbers in the posterior part of the body cavity (Becker and Flammang, 2010). These organs are expelled as a defence mechanism when the sea cucumber is disturbed, for example by a potential predator (Fig. 1E). In *H. forskali*, Cuvierian tubules consist of, from the inside to the outside, an inner epithelium surrounding the narrow lumen, a thick connective tissue layer and an outer mesothelium (Fig. 9A). The mesothelium is the tissue layer responsible for adhesion (VandenSpiegel and Jangoux, 1987; Demeuldre et al., 2014). In quiescent tubules, it is a pseudostratified epithelium made up of two superimposed cell layers: an outer layer of peritoneocytes and an inner layer of granular cells which is highly folded along the long axis of the tubule (Fig. 9B,C). At this level, only the mucus vacuoles of peritoneocytes and the connective tissue present in-between the granular cells were stained with Alcian Blue, at both pH 1 and 2.5 (Fig. 9A-C). The reactivity to Alcian Blue was lost after methylation, but not restored when methylation was followed by saponification, indicating that this reactivity was due to the presence of sulfated macromolecules (Table 2). This staining pattern confirms the observations of Guislain (1953) on the mesothelium of the Cuvierian tubules of *Holothuria impatiens*. With the anti-sulfotyrosine antibodies, only the mucus vacuoles enclosed in the peritoneocytes were labelled (Fig. 9D).

**Fig. 10.**
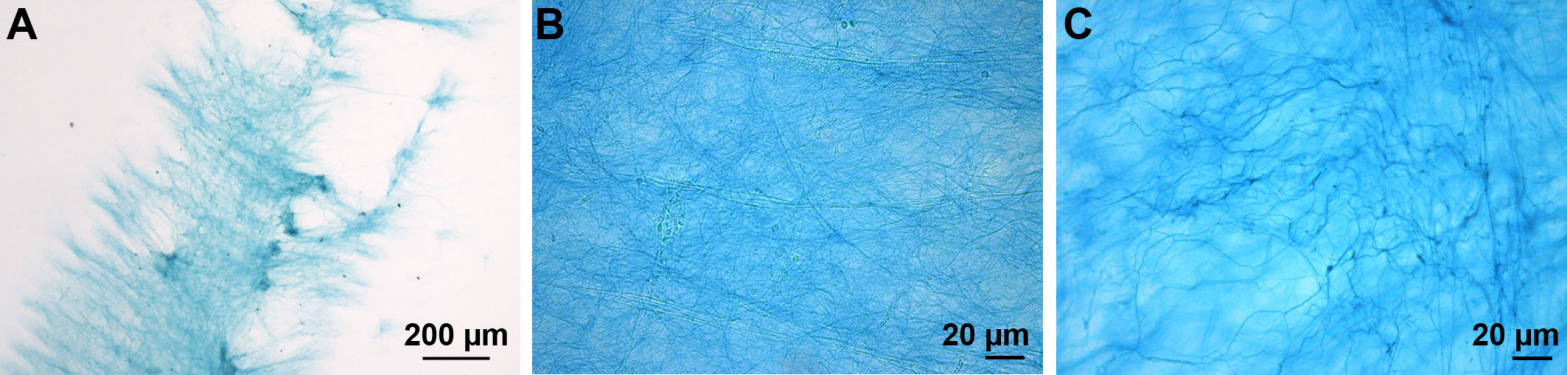
**Cuvierian tubule prints of *H. forskali* stained with Alcian Blue.** (A) General view of a tubule print at pH 1. (B,C) Details of the print material at pH 1 (B) and pH 2.5 (C).

Cuvierian tubule prints comprise the adhesive material released by the granular cells but also collagen fibres originating from the tubule connective tissue layer (Demeuldre et al., 2014). These prints were stained with Alcian Blue at both pH 1 and 2.5, although the staining was more intense at pH 2.5 (Fig. 10; Table 2). No stain was observed with Alcian Blue at pH 2.5 following methylation, while the reactivity was partially restored when methylation was followed by saponification (Table 2). A strong immunolabelling was observed for the whole prints with the anti-sulfotyrosine residues. However, an identical labelling was also observed for the controls, in which only the secondary antibodies were applied to the prints, indicating an aspecific labelling of the tubule prints.

## DISCUSSION

Sulfate moieties are one of the chemical groups proposed to be involved in the underwater adhesion of marine organisms (Stewart et al., 2011; Petrone, 2013). However, algae are the only organisms in which the involvement of sulfates in the adhesion process has been demonstrated through spectroscopic investigations of their secreted adhesive holdfast (Chiovitti et al., 2008; Petrone et al., 2011; Dimartino et al., 2016). In metazoans, the occurrence of sulfate groups in the adhesive material generally remains speculative because, in many cases, they have only been detected in gland cells by histochemical methods. Using a colorimetric assay, we quantified the sulfate content of adhesive secretions from three marine invertebrate species: the mussel *M. edulis*, the sea star *A. rubens* and the sea cucumber *H. forskali*. In these adhesive materials, sulfates amounted for about 1% of the glue dry weight. These values are much lower than the 17% reported in the literature for the limpet *P. vulgata* (Grenon and Walker, 1980). In the glue of the terrestrial slug *A. subfuscus*, the sulfate content is about 6% (Braun et al., 2013).

The presence of sulfated macromolecules was also highlighted using both Alcian Blue staining and anti-sulfotyrosine antibodies in the adhesive organs and adhesive secretions of mussels, tubeworms, sea stars, limpets and sea cucumbers. No sulfated biopolymers were detected in the cement glands of the tubeworm *S. alveolata*, unlike what has been reported for the closely related species *P. californica* (Wang and Stewart, 2013). Such molecules were detected, however, in the four other species.

Different types of secreted macromolecules, such as proteins, glycoproteins, proteoglycans and polysaccharides can undergo sulfation (Hemmerich, 2007; Pomin, 2009). This chemical modification involves the transfer of a sulfate moiety to tyrosine residues in the case of proteins, or to carbohydrates in glycoproteins, proteoglycans and polysaccharides, generating so-called sulfotopes (Hemmerich, 2007). Whereas proteins and glycoproteins generally bear one or a few distinct sulfotopes per molecule, proteoglycans and polysaccharides harbor many, frequently clustered sulfotopes per molecule (Hemmerich, 2007). This is the reason why it is generally admitted that the cationic dye Alcian Blue (at pH 1) stains only sulfated proteoglycans and polysaccharides that bear a high density of negative charges (Bancroft and Gamble, 2001). Anti-sulfotyrosine antibodies, on the other hand, specifically label sulfated proteins and possibly glycoproteins (Medzihradszky et al., 2004; Hemmerich, 2007). Therefore, the two methods presumably highlight different biopolymers although common staining and labelling of the same molecule cannot be excluded. Our results appear to corroborate this hypothesis. Indeed, when the adhesive organs are positively stained with both methods, the two types of labelling either do not co-localize (e.g. in sea stars) or, if they do, they are presumably indicative of different molecules (e.g. in mussels). In *A. rubens*, Alcian Blue stains the adhesive cells while anti-sulfotyrosine antibodies label another, non-adhesive cell type. As expected, therefore, the adhesive footprints are positive only to Alcian Blue. In *M. edulis*, three groups of mucous cells (anterior, posterior and lateral) were highlighted that surround the groove and depression in which byssal threads and plaques are produced. All these cells are positive with both methods but the byssus is stained exclusively with the Alcian Blue method. This suggests that the sulfated carbohydrate-containing molecules would be released and incorporated into the byssus whereas the sulfotyrosine-containing proteins would not be secreted. Similarly, in the Cuvierian tubules of *H. forskali*, the contents of the mucous vesicles from peritoneocytes is labelled with both Alcian Blue and anti-sulfotyrosine antibodies but the adhesive print material stains only with the former. Finally, in *P. vulgata*, no sulfotyrosine residue was detected. Nevertheless, most pedal glands and the secreted adhesive material are extensively stained with Alcian Blue.

As exemplified by the five biological models investigated in the present study, marine invertebrate adhesive systems may differ considerably, allowing to define four types of adhesion: permanent, temporary, transitory and instantaneous. Although both permanent and instantaneous adhesion are clearly unique, the boundary between transitory and temporary adhesion is not always clear. Indeed, some gastropod molluscs may alternate between both types of adhesion (Smith et al., 1999). Moreover, all non-permanent adhesives, temporary and transitory, share similar amino acid compositions, even among very disparate organisms (Flammang, 2006). Similarly, they may also share a potential involvement of sulfate groups in their adhesion process. Indeed, among the species investigated in the present study, the sea star *A. rubens* and the limpet *P. vulgata* present the highest sulfate content in their secreted adhesive material. Moreover, the strong Alcian Blue staining of this material as well as of the cells that produce it indicates it presumably contains sulfated proteoglycans or polysaccharides.

Sulfated macromolecules have also been detected in the adhesive secretions of mussels and sea cucumbers but, for these two species, functions other than adhesion are more likely. In the Cuvierian tubules of *H. forskali*, the contents of peritoneocyte mucous vesicles is apparently not incorporated in the adhesive material (Demeuldre et al., 2014). When tubules are expelled and elongate, these cells disintegrate, with their mucus content being released. This mucus would cover the tubule outer surface, supposedly acting as a protective coating that prevents elongating tubules from adhering to each other and to the holothuroid body (VandenSpiegel and Jangoux, 1987). As for the sulfate content of the adhesive prints, it probably derives from a contamination of the adhesive material with connective tissue during peeling (Demeuldre et al., 2014). This tissue layer is indeed stained with Alcian Blue at pH 1. Moreover, when this contaminant material is partly removed by a first light scraping, the sulfate content of the remaining glue-enriched prints is halved. Mussel is the best studied marine organism in terms of adhesion (see e.g. Waite, 2017; for review) but sulfate groups have never been reported to take part in their adhesion mechanism. In *M. edulis*, the presence of Alcian Blue positive mucous cells all around the foot groove and the localization of their secretion in the outer cuticle of the byssal thread and plaque suggest that sulfated biopolymers could also have an anti-adhesive function. Their role would be to prevent the forming byssal thread from adhering to foot tissues and therefore to facilitate its disengagement from the groove. Moreover, a high density of sulfate groups could account, at least partly, for the low pH recorded within the closed space created within the groove and under the distal depression. This low pH favours adhesion by triggering the adsorption of catechol groups from byssal proteins to surfaces (Waite, 2017). Alternatively, the secretions from the anterior mucous cells might also be involved in the non-permanent attachment of the foot tip to the substratum during byssus secretion (Hwang et al., 2010).

In terms of adhesive mechanisms, sulfated macromolecules thus appear to play a role only in non-permanent adhesion. In comparison with catechol or phosphate functionalities found in permanent adhesives, sulfates possess weaker coordination ability and do not adsorb strongly to mineral surfaces, especially at the pH of seawater (Petrone, 2013). Yet, organisms such as sea stars and limpets display an adhesion strength almost as high as that of organisms using permanent adhesion (Flammang et al., 2016). This high tenacity could then be mediated by the cohesive role of sulfated macromolecules. In the sea star *A. rubens*, the footprint meshwork which forms the structural scaffold of the adhesive material stains with Alcian Blue at pH 1. At this level, sulfated polysaccharides could interact with the carbohydrate-binding domains of Sfp1 (Hennebert et al., 2014). In limpets and other gastropod molluscs, it has been suggested that large sulfated macromolecules such as proteoglycans and glycosaminoglycans entangle to form the viscoelastic core of the pedal mucus (Smith, 2016b). It is also the case for the slug defensive secretions in which the tangled network of proteoglycans is essential to the toughness of the glue by allowing extensive deformation before fracture (Wilks et al., 2015). In ECM proteoglycans, sulfates are binding sites for adhesion domains of structural proteins and these interactions promote extracellular matrix assembly and govern its physical properties (Hemmerich, 2007; Lindahl et al., 2017). Thus, in non-permanent adhesives, the distribution of sulfated proteoglycans or polysaccharides correlates well with a cohesive function at the level of the bulk of the adhesive material.

## MATERIALS AND METHODS

### Animal collection and maintenance

Five marine invertebrates, representative of different types of adhesion, were included in the present study (Fig. 1). Mussels (*M. edulis* Linnaeus, 1758), sea stars (*A. rubens* Linnaeus, 1758) and limpets (*P. vulgata* Linnaeus, 1758) were collected intertidally at Audresselles (Pas-de-Calais, France). Honeycomb worms (*S. alveolata* Linnaeus, 1767) were sampled from the Champeaux reef located in the eastern part of Mont-Saint-Michel Bay (Manche, France). Sea cucumbers (*H. forskali* Delle Chiaje, 1823) were obtained from the Observatoire Océanologique of Banyuls-sur-Mer (Pyrénées- Orientales, France). All individuals were kept in marine aquaria with closed circulation [18°C, 33 practical salinity units (psu) for sea cucumbers; 13°C, 33 psu for mussels, sea stars, limpets and tubeworms]. Animals used in our experiments were maintained and treated in compliance with the guidelines specified by the Belgian Ministry of Trade and Agriculture.

### Sulfate quantification

Adhesive material from *M. edulis*, *A. rubens*, and *H. forskali* was collected as follows. Individuals of *A. rubens* were allowed to walk across and/or attach to the bottom of clean glass Petri dishes filled with filtered sea water for 8 h. For *H. forskali*, the discharge of the Cuvierian tubules was induced mechanically by pinching the dorsal integument of sea cucumbers with forceps. The expelled tubules were collected in Petri dishes filled with seawater. After the tubules adhered firmly on the bottom of the Petri dishes, their collagenous cores were detached manually using fine forceps. All the Petri dishes were then thoroughly rinsed in ultra-pure water and freeze-dried. The lyophilized adhesive material was then scraped off using a razor blade (Flammang et al., 1998; De Moor et al., 2003). For sea cucumber Cuvierian tubules, two types of materials were collected: whole prints obtained as described above, and glue-enriched prints obtained by first scraping lightly the dishes and discarding this material and then scraping again more strongly. Individuals of *M. edulis* were allowed to attach overnight to clean Petri dishes filled with seawater. The byssal threads were cut using a scalpel and the attached adhesive plaques were then scrapped off from the Petri dishes. They were collected, rinsed in ultra-pure water and freeze-dried. Total sulfate content of each adhesive material was assayed by the benzidine method, as modified by Antonopoulos (1962). The calibration curve for this assay was established using K_2_SO_4_ and the absorbance values were taken from the linear range, where the measurement error was roughly 5%.

### Histochemical and immunohistochemical analyses

Feet and byssal plaques of *M. edulis*, anterior parts of *S. alveolata*, tube feet of *A. rubens*, feet of *P. vulgata* and Cuvierian tubules of *H. forskali* were all fixed in Bouin's fluid, dehydrated in graded ethanol, embedded using a routine method in paraffin wax (Gabe 1968), and sectioned at a thickness of 5 µm with a Microm HM 340E microtome. A few sections were stained with Arnow's method to highlight DOPA-containing proteins (Arnow, 1937). Adhesive footprints from *A. rubens* and *H. forskali* were collected as described above, but on microscope glass slides. To collect footprints from *P. vulgata,* one limpet was allowed to attach to clean microscope glass slides placed on the bottom of a Petri dish filled with filtered seawater. All the footprint-covered slides were fixed in Bouin's fluid and stored in 70% ethanol.

#### Alcian Blue staining

Histological sections and adhesive footprints were stained with 1% (w/v) Alcian Blue 8GX in 0.1N HCl (pH 1) or with 0.5% (w/v) Alcian Blue 8GX in 3% (v/v) acetic acid (pH 2.5). Furthermore, for more accurate interpretation of the results, the pH 2.5 condition was also applied to sections and footprints which had been pre-treated by methylation [1% (v/v) HCl in methanol, 5 h at 60°C], and methylation followed by saponification [1% (w/v) KOH in 70% (v/v) ethanol, 30 min at room temperature] (Bancroft and Gamble, 2001). Sections and footprints were observed using a Zeiss Axioscope A1 microscope equipped with an AxioCam ICc3 camera. Images were acquired using the Zeiss AxioVision 4.7 software.

#### Immunohistochemistry

Histological sections and adhesive footprints were subjected to an indirect immunohistochemical staining method according to the following protocol. Antigen retrieval was achieved by incubation in a solution containing 0.05% (w/v) trypsin (Sigma-Aldrich) and 0.1% (w/v) CaCl_2_ for 15 min at 37°C. The sections were then washed for 3 min in water. The antigen retrieval step was not performed on footprints. Sections and footprints were blocked for 30 min in Tris-buffered saline containing 0.05% (v/v) Tween 20 and 3% (w/v) BSA (TBS-T-BSA). Monoclonal anti-sulfotyrosine antibodies (clone Sulfo-1C-A2, Merck Millipore) diluted 1:100 in TBS-T-BSA were applied to the sections and footprints for 2 h at room temperature. After three washes of 5 min in TBS-T, the sections and footprints were incubated for 1 h in Alexa Fluor 568-conjugated goat anti-mouse immunoglobulins (Life Technologies) diluted 1:100 in TBS-T-BSA. Following three final washes of 10 min in TBS-T, the sections and footprints were mounted in Vectashield (Vector Laboratories). Control reactions were performed by substituting the primary antibodies with TBS-T-BSA and/or by pre-treating sections using the methylation and saponification treatments described above. Sections and footprints were observed using the Zeiss Axioscope A1 microscope. Some tube foot sections from *A. rubens* were also submitted to a co-labelling with antibodies directed against the adhesive protein Sfp1 (anti-VDGNDFEYITDEDGRD diluted 1:100; Hennebert et al., 2014). In that case, the Alexa Fluor 568-conjugated goat anti-mouse immunoglobulins were mixed with Alexa Fluor 488-conjugated goat anti-rabbit immunoglobulins (Invitrogen). These sections were observed using an Olympus Fluoview fv1000 confocal microscope.
